# Socioeconomic gradients in the effects of universal school-based health behaviour interventions: a systematic review of intervention studies

**DOI:** 10.1186/s12889-015-2244-x

**Published:** 2015-09-17

**Authors:** Graham F. Moore, Hannah J. Littlecott, Ruth Turley, Elizabeth Waters, Simon Murphy

**Affiliations:** DECIPHer, School of Social Sciences, Cardiff University, 1-3 Museum Place, Cardiff, CF10 3BD UK; Jack Brockhoff Child Health and Wellbeing Program, School of Population and Global Health, University of Melbourne, Melbourne, Australia

## Abstract

**Background:**

Socioeconomic inequalities in health behaviour emerge in early life before tracking into adulthood. Many interventions to improve childhood health behaviours are delivered via schools, often targeting poorer areas. However, targeted approaches may fail to address inequalities within more affluent schools. Little is known about types of universal school-based interventions which make inequalities better or worse.

**Methods:**

Seven databases were searched using a range of natural language phrases, to identify trials and quasi-experimental evaluations of universal school-based interventions focused on smoking, alcohol, diet and/or physical activity, published from 2008–14. Articles which examined differential effects by socioeconomic status (*N =* 20) were synthesised using harvest plot methodology. Content analysis of 98 intervention studies examined potential reasons for attention or inattention to effects on inequality.

**Results:**

Searches identified approximately 12,000 hits. Ninety-eight evaluations were identified, including 90 completed studies, of which 20 reported effects on SES inequality. There were substantial geographical biases in reporting of inequality, with only 1 of 23 completed North American studies testing differential effects, compared to 15 out of 52 completed European studies. Studies reported a range of positive, neutral or negative SES gradients in effects. All studies with a negative gradient in effect (i.e. which widened inequality) included educational components alone or in combination with environmental change or family involvement. All studies with positive gradients in effects included environmental change components, alone or combined with education. Effects of multi-level interventions on inequality were inconsistent. Content analyses indicated that in approximately 1 in 4 studies SES inequalities were discussed in defining the problem or rationale for intervention. Other potential barriers to testing effect on inequality included assumptions that universal delivery guaranteed universal effect, or that interventions would work better for poorer groups because they had most to gain.

**Conclusions:**

Universal school-based interventions may narrow, widen or have no effect on inequality. There is a significant need for more routine testing of the effects of such interventions on inequality to enable firmer conclusions regarding types of interventions which affect inequality.

**PROSPERO registration number:**

CRD42014014548

**Electronic supplementary material:**

The online version of this article (doi:10.1186/s12889-015-2244-x) contains supplementary material, which is available to authorized users.

## Background

Many indicators of health, including disability-free life years and life expectancy, are positively associated with socioeconomic status (SES) [1]. While socioeconomic inequalities in health cannot be fully explained by behavioural factors [2], behaviours such as smoking and diet play a role [3, 4]. Inequalities in health behaviours emerge during childhood [5], while recent international evidence indicates that these inequalities are widening, in line with growing economic inequality [6].

Attempts to improve young people’s health behaviours commonly involve school-based interventions, often targeting lower SES pupils or schools [7]. While intuitively appealing, targeting usually ignores the graded nature of associations between socioeconomic status and health [1] and may produce stigma [8], while imprecise targeting methods often fail to reach at-risk individuals. Targeting deprived schools for example may fail to reach poorer children in more affluent schools, or address inequalities within such schools [9]. Hence, universal interventions which disproportionately benefit lower SES groups may have greater potential to improve population health, while reducing inequality [1]. However, depending on the nature of intervention, universal approaches may also worsen inequalities [10, 11].

McLaren and colleagues [11] argue that the likelihood of universal interventions worsening or improving inequalities depends on whether intervention involves ‘superficial’ (i.e. focusing upon individual agency) or ‘radical’ change (i.e. targeting structural factors). Some empirical evidence lends support to notions that ‘downstream’ interventions are more likely to worsen inequality [12]. Interventions targeting individual factors such as knowledge may work where ignorance is the sole reason for not ‘choosing’ healthier options, though may do little for individuals facing greater structural constraints [13]. However, social and environmental interventions may be resisted where incongruent with local norms and reducing inequality may require intervention at multiple levels [14, 15]. Some reviews indicate that school-based interventions based on education are often ineffective [16], while multi-level interventions have greater effects [17]. One would perhaps hypothesise that school-based interventions based purely upon education may be more likely to worsen inequalities, while interventions targeting factors at multiple levels simultaneously, may be more likely to reduce inequality.

However, evaluations have traditionally paid little attention to effects on inequalities; in a 2013 Cochrane review of school-based interventions to prevent adolescent smoking, socioeconomic inequalities are not mentioned [16]. In a 2013 review of physical activity interventions, none of the 44 trials reviewed examined effects upon inequalities [18]. In a 2014 review of the WHO Health promoting schools framework [19], 2 of 67 included studies reported effects by SES. A recent review of equity impacts of tobacco control policies, identified 5 childhood studies which reported effects by SES [20], including 2 in a school setting. One study reanalysing European trials of ‘effective’ interventions in four behavioural domains (smoking, diet, physical activity and alcohol use) reported that effects often varied by SES, but drew few conclusions regarding why due to the small number of studies [21–24].

Inattention to sub-group effects may in part reflect tensions between needs of policymakers and practitioners, and a desire for scientific purity. Sub-group analysis, while critical for policy and practice, is often viewed as statistical malpractice, with studies typically powered to detect a main effect [25]. It may also reflect assumptions that universal delivery ensures movement of the risk distribution to the left, with no effect on inequality [26]. Schools are commonly seen as useful venues for health improvement precisely because they may reach almost all children. However, interventions do not ‘work’ upon passive recipients, but are interacted with in ways which are shaped by pre-existing contextual conditions [27, 28]. Inattention to inequality may also reflect utilitarian approaches, focused on achieving the greatest gain for the greatest number. Recent discourses surrounding inequalities have attempted to highlight economic consequences for society as a whole [29], while others maintain that addressing health inequalities remains a moral issue. Intervention developers’ and evaluators’ views on the moral and economic importance of inequality, or dominant discourses within their countries may shape the nature of interventions developed or evaluated, and decisions on whether effects on SES should be tested.

In 2007, Whitehead [30] called for all interventions which aim to improve population health to be evaluated in terms of effects on inequality. This review examines the extent to which, and the ways in which effects of universal school-based interventions on socioeconomic inequality have been evaluated in peer-reviewed evaluations published since 2008. Authors such as McIntyre have highlighted the fallibility of assuming all measures of SES to be equal [31], with socioeconomic trends differing by means of measurement (for example, whether assessing income or education level). Hence, the types of SES markers collected will also be summarised. A secondary aim is to synthesise existing evidence on the types of interventions which are more likely to worsen or improve inequality, although conclusions will be drawn tentatively due to the shortage of available studies. No previous reviews to date have examined how socioeconomic inequalities are discussed within evaluation studies, in order to gain insights into why an inequality focus features so infrequently. Hence, we report a content analysis of discussion of socioeconomic inequality within the rationale for interventions and interpretation of findings within published articles of school-based interventions.

## Methods

### Inclusion criteria

A sensitive search strategy was applied using a broad range of natural language phrases (see Additional fileS 1, 2 and 3) in Medline, Psychinfo, EMBASE, ASSIA, British Education Index, Sociological abstracts and ERIC. The search was conducted in April 2014 and limited to studies reporting main outcomes since 2008 and available in the English language. Randomised controlled trials, or quasi-experimental studies (studies with a non-randomly allocated comparison group), were included. The following inclusion criteria were applied:

*Population* - school children (age 4–18). The review includes universal interventions (i.e. aimed at whole school or whole year groups). It excludes interventions targeted toward special populations (e.g. children with special educational needs or health conditions), which are not delivered to the general school population or for which schools were selected on the basis of high risk demographic profiles (e.g. lower SES schools only).

*Interventions* - interventions delivered partially or wholly within the school setting, or relating to travel to school.

*Control* - no intervention or practice as usual. Studies focused on relative effectiveness of two active interventions without a control group were excluded.

*Outcomes* - impacts on diet, physical activity (including measures of physical fitness), smoking or alcohol. Studies were included if they measured one or more of these behaviours. Small pilot studies, efficacy studies (e.g. studies evaluating impact of specific forms of physical activity on fitness parameters) or studies including fewer than 10 clusters (i.e. schools or classes) were excluded.

*Setting* - Schools

### Screening

Identified studies were imported into Reference Manager. Titles and abstracts were double screened, with disagreements resolved through discussion between researchers. Full-texts were obtained and screened for eligibility by both researchers, with disagreements resolved through discussions. Reasons for exclusion were recorded for all studies excluded at the full text stage.

### Additional searches for linked publications

Forward citation tracking in Google Scholar was used to identify subsequent papers from the study which made reference to the identified protocol/outcomes papers. Process evaluation articles linked to outcomes studies which *did* examine effects by SES were obtained in order to examine the use of process evaluation to understand inequalities in the effects of the intervention.

### Data extraction

For each study retained after screening, 2 researchers completed data extraction independently in duplicate. These captured study name and date, design, methodological parameters (e.g. sample size), intervention type (whether comprising: education, social/physical environmental change, family/community involvement), outcome measures, country of origin, details of SES measures collected and how these were used, as well as any other discussion of SES inequality. [31] For articles which reported effectiveness by SES, subgroup effects or interaction effects were extracted from the articles.

### Synthesis

Proportions of eligible studies collecting SES measures and how these were used was quantifed (e.g. the number of studies using measures for sample descriptions, as confounders). Numbers and percentages which analysed differential effects (by intervention type, geographical region, behavioural outcome and study year) were quantified. All studies which presented subgroup effects or interactions by SES were assessed by 2 coders using a standardised quality assessment tool for quantitative studies (http://www.ephpp.ca/PDF/Quality%20Assessment%20Tool_2010_2.pdf). Effects on inequality were sub-divided by intervention and outcome type, geographical region, sub-group measurement type (i.e. family level measures such as parental education, occupation or income, or school-level measures such as free school meal entitlement) and presented using harvest plot methodology [32]. Consistent with Project TEENAGE [24], we included measures of parental education, occupation or income, as well as school-level measures such as area deprivation or free school meal entitlement levels. Effects of interventions were separated by school and family level measure, although no further disaggregation was possible with this number of studies. A content analysis of the manuscripts texts for all 98 identified was used to categorise the prevalence and nature of talk about socioeconomic inequalities. Two reviewers (GFM & HJL) independently read each article and extracted sections discussing SES inequality, copying these to a table. An a priori framework for coding discussion of inequality represented by this table, (with headings including whether inequalities were discussed in defining the problem, whether the authors stated reduction in inequality as an intervention aim) was refined in discussion between reviewers, before being applied to all included studies.

## Results

### Description of studies obtained

**Fig. 1:**
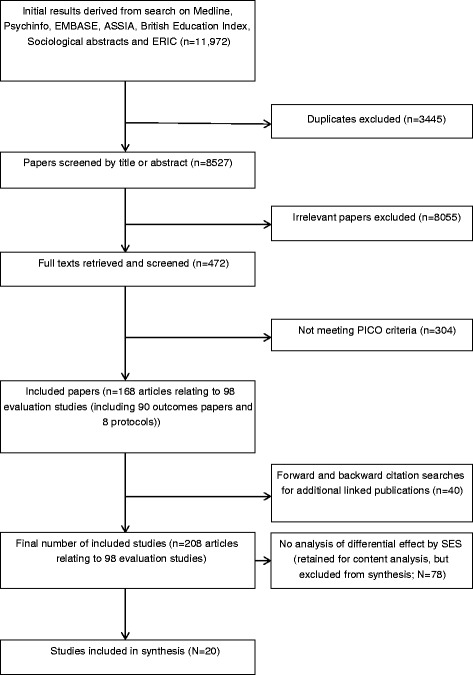
Flow diagram of study selection into the review

Study selection and inclusion are indicated in Fig. 1. The search identified 11,972 hits, which after removal of duplicates, reduced to 8527. Full texts were retrieved for 472 articles, of which 168 (related to 98 studies – see additional files 1, 2 and 3) met inclusion criteria. Forward and backward citation searches for linked publications identified 40 additional articles. Outcomes analyses had been published for 90, while 8 were protocols for studies yet to report outcomes. Most were from Europe (*n =* 58) or North America (*N =* 24), with 9 from Australasia, 4 from South America and 3 from Asia. The most common intervention types were education only (*n =* 30), ‘multi-level’ interventions combining education, environment/ethos (i.e. attempts to modify the social and physical environment of the school setting) and family/community involvement (*n =* 27) or interventions focused on education and environment/ethos (*n =* 16). Eleven focused on education and family involvement, 7 on environment/ethos and family involvement, 6 on environment/ethos alone and 1 on family/community involvement alone. Due to small numbers in some groups, for subsequent analyses, studies are grouped as ‘education only’, ‘environmental change’ and ‘education and environmental change’. Twenty focused exclusively on physical activity, 19 on diet and 21 on both. Fifteen focused on tobacco, 12 alcohol and 11 both.

### Measures of SES and their usage

The numbers of studies which collected measures of SES, and how these were used within the evaluation, are presented in Table 1. Most studies (71 out of 98) collected a measure of SES, with these most commonly simply presented in sample descriptions. Of the 90 completed studies, 19 reported analysis of effects by SES [33–52]. Of studies yet to report outcomes, 2 of 8 reported plans to analyse effects on inequality [53, 54]. Differential effects were sometimes reported in separate articles or across multiple papers [55–61]. One additional study was reanalysed as part of project TEENAGE [24], providing a total of 20 studies for synthesis. Although the collection of measures of SES has become substantially more common in more recent studies (from 26/42 earlier studies to 45/56 more recent studies), their use to evaluate differential effects has increased very little. Analysis of effects by SES also varied by behavioural outcome and region; for example, only 1 out of 23 completed North American studies examined effects on inequality, 16 out of 52 European studies did so. Of studies for which only protocols were identified, both which stated that they would examine effects by SES were European. Thirty-one studies examined differential effects by other key demographics including sex (30 studies) and ethnicity (6 studies).

**Table 1:** Collection and uses of measures of socioeconomic status within intervention trials overall, and by year, region and behavioural outcome

		All studies (*n =* 98)	Study year (completed studies)		Region					Behavioural outcome					
			2008-2010 (*N =* 42)	2011-2014 (*N =* 56)	Europe (*N =* 58)	N America (*N =* 24)	Australasia (*N =* 9)	Asia (*N =* 3)	S America (*N =* 4)	Diet (*N =* 19)	Physical activity (*N =* 20)	Diet and PA (*N =* 21)	Smoking (*N =* 15)	Alcohol (*N =* 12)	Smoking and alcohol (*N =* 11)
Measure collected	Any measure of SES	71	26	45	46	15	7	1	2	14	14	16	13	8	6
	School level (e.g. Free School Meals)	32	12	20	14	11	6	1	0	7	7	5	6	3	4
	Family level (e.g. parental income)	48	18	30	37	7	3	0	2	8	10	11	9	6	5
Use of SES measures	Sample descriptions	53	22	31	34	12	4	1	2	11	9	15	4	6	4
	Control variables	24	8	16	16	3	4	0	1	6	4	6	5	2	1
	Sampling procedures	19	9	10	9	5	4	1	0	4	4	3	3	3	2
	Analysis of differential effect	21^a^	8	13	18^a^	1	1	1	0	4	1	8	4	2	2

^a^includes 2 protocols for which analysis not yet published – not included in synthesis

### Effects on inequality

**Fig. 2:**
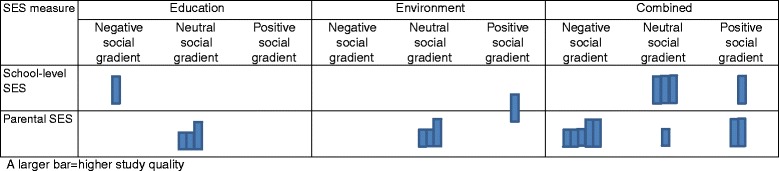
Harvest plot for intervention effects by SES inequality

Details of interventions and effects of the twenty studies for which effects on inequality are presented in Table 2 and assessments of their quality in Table 3. Most were multi-level interventions, with only 4 education-only interventions testing effects on inequality. Studies were of moderate (*N =* 12) to low quality (*N =* 8), the most common weakness being low school-level response rates. Approximately 2 in 3 (*N =* 12) studies described being informed by 1 or multiple theoretical frameworks. These were almost exclusively drawn from social psychology, with the most commonly referenced frameworks being Social Cognitive Theory (*N =* 5), the Theory of Planned Behaviour (*N =* 3) and Self-efficacy Theory (*N =* 2). None related these frameworks to mechanisms for addressing inequality. Methods for testing differences in effect varied, with 12 reporting testing interaction effects only, 2 subgroup analysis only and 6 both. Most studies reporting a non-significant interaction did not report test statistics. While some conducted tests of differential effect for all outcomes, others tested effects on selected outcomes (e.g. outcomes which had shown a significant main effect, or primary outcomes only). Overall, 10 studies reported no social gradient in effectiveness, 6 a negative social gradient in at least one outcome, and 4 a significant positive gradient for at least one outcome (Fig. 2).

**Table 2:** Description of included interventions and effects

Study name	Main reference	Country	SES measure^a^	Intervention type	Outcomes tested	Effective?	Outcomes analysed bv SES	Gradient in effect	
AFLY5	Kipping et al. 2014	UK	School IMD Pupil IMD	Education and parental involvement	Physical activity (accelerometer) and diet outcomes	No effects on primary outcomes. Significant change in 3 secondary outcomes.	Physical activity (accelerometer) and diet outcomes	Some subgroup differences in both directions (e.g. effect on snacking only in low SES, and on central obesity only in high SES). No significant interaction effects.	Neutral ^e^
ASSIST	Campbell et al. 2008	UK	FSM FAS	Education and environment	Smoking status	Significantly lower rise in smoking rates in intervention group.	Smoking status	Neutral (OR for interactio*n =* 0.99) ^c^	Neutral
School fruit scheme	Bere et al. 2010	Norway	Parental education	Environment	Fruit consumption and vegetable consumption	Significant increases in fruit intake – no change in vegetable intake	Fruit consumption and vegetable consumption	Neutral (statistics not reported)	Neutral
Crone et al. 2011	Crone et al. 2011	Netherlands	Parental education and student education level	Education	Smoking	Increased intention not to smoke and lower smoking uptake after transition to secondary school.	Smoking	Neutral (statistics not reported)	Neutral
EHealth4us	Bannink et al. 2014	Netherlands	Parental education, employment and family affluence	Education	Smoking and alcohol (as secondary measures – primary outcomes mental health)	No significant main effects	All outcomes	Neutral (statistics not reported)	Neutral
Energize	Rush et al. 2012	New Zealand	School deprivation decile	Education, environment and family/community involvement	Diet and physical activity (as secondary outcomes – primary outcomes obesity measures)	No significant main effects	Obesity and blood pressure only	Larger effects on BP and body-fat in more affluent schools	Negative
ESFA	Ariza et al. 2008	Spain	Parental education and ‘family economic capacity index’. Neighbourhood SES	Education, environment and family/community involvement	Smoking	Significantly lower rate of increase in smoking in experimental group.	Smoking	Bigger effect in high FECI. No clear difference by parental education	Negative
EUDap	Faggiano et al. 2010	Austria, Belgium, Germany, Greece, Italy, Spain, Sweden	Area (school) level SES measure	Education, environment and family/community involvement	Smoking and alcohol (plus cannabis and other drugs)	Significant effects found for daily cigarette smoking and episodes of drunkenness in the past 30 days for at least one episode, or three or more episodes..	Alcohol only	Larger effects on alcohol consumption measures in more deprived schools.	Positive
FatAintPhat	Ezendam et al. 2012	Netherlands	School type (vocational or pre-university)	Education	Physical activity, sedentary behaviour, fruit and vegetable intake, snacking and sugar sweetened beverages (plus BMI, waist circumference and fitness)	No effect on primary outcomes (BMI). But positive effect on some secondary outcomes (fruit and vegetable intake, snacking and sugar sweetened beverages). Negative effect on step counts.	Fruit and vegetable intake, snacking and sugar sweetened beverages (only variables with a significant and + ve main effect)	Effect on SSB only in higher SES schools. No other significant interactions	Negative
HEIA	Grydeland et al. 2012	Norway	Parental education	Education, environment and family/community involvement	Physical activity (accelerometer) and dietary outcomes (plus obesity outcomes)	Mixed	All outcomes	Greater effect on BMI for higher SES. No interactions for behaviours.	Negative
KOPS	Plachta-Danielzik 2011	Germany	Parental education	Education, environment and family/ community involvement	Healthy eating index, physical activity and media time (as secondary outcomes – primary outcomes obesity measures)	No significant main effects	All outcomes	Bigger effect on BMI for higher SES. No interactions for behaviours.	Negative
Avall	Llargues et al. 2011	Spain	Mother’s/Father’s education	Education, and family/community involvement	Physical activity and diet (as secondary outcomes – primary outcomes obesity measures)	Lower rise in BMI in intervention group. Twenty dietary and physical activity secondary outcomes tested.	BMI only	Effects on BMI only in high SES	Negative
STOPP	Marcus et al.	Sweden	Parental education	Education, environment and family/community involvement	Physical activity and diet (as secondary outcomes – primary outcomes obesity measures)	Effects on BMI among those who were overweight at baseline only. Mixed effects on 8 secondary diet outcomes.	Diet outcomes	Bigger effect on dairy product and fast food intake in low SES.	Positive
MYTRI	Perry et al. 2009	India	School type (government vs private)	Education, environment and family/community involvement	Smoking	Lower increases in smoking or bidi uptake in intervention group.	Smoking	Neutral (statistics not reported)	Neutral
PAS	Koning et al. 2009	Netherlands	Parental education and school type (vocational vs academic)	Education, environment and family/community involvement	Alcohol use (Heavy weekly, weekly and frequency)	At first follow-up, only the combined student–parent intervention showed substantial and statistically significant effects on heavy weekly drinking, weekly drinking and frequency of drinking. At second follow-up these results were replicated, except effects on heavy weekly drinking.	Alcohol use (WD and HWD)	Bigger effect on HWD in low-educated adolescents. No moderation of effect on WD.	Positive
Promise	Stallard et al. 2012	UK	Family affluence	Education	Alcohol	No effects on primary outcome, or substance use (measured as secondary)	Mental health outcomes only	Neutral (OR for interactio*n =* −0.45(−1.11 to 0.21))	Neutral
PSFBI	Murphy et al. 2011	UK	School and individual FSM	Environment	Diet (breakfast skipping and healthy/unhealthy items)	Significant improvements in diet quality at breakfast and attitudes toward breakfast. No differences in breakfast skipping, fruit and veg intake or sweets and crisps.	All outcomes	Bigger effect in low SES for breakfast skipping and healthy breakfast items. No other significant interactions. ^c^	Positive
Smart Lunchbox	Evans et al. 2010	UK	FSM	Environment and family/community involvement	Diet outcomes	Intervention group children were provided with more fruit, vegetables, dairy food and starchy food other than bread. Weight of savoury snacks (crisps and other salted snacks) lower for children in the intervention group. Weights of sweetened drinks and confectionery did not change	All outcomes	Neutral (statistics not reported)	Neutral
SPACE	Toftager et al. 2014	Denmark	Income and ‘parental SES’	Environment	Physical activity, fitness, active transport and obesity	No significant main effects	All outcomes except active travel	Neutral (statistics not reported)	Neutral
Pro Children ^d^	Te Velde et al. 2008	Netherlands, Spain and Norway	Parental education	Education, environment and family/community involvement	Diet outcomes	Significant effects for fruit and veg intake found at first follow-up. One year later, a significant impact was only observed in Norway.	All outcomes	Non-significant interaction effects (data unreported) Effects on F&V in high and low SES.	Neutral

^a^Bolded item is item used for analysis of differential effects where multiple SES measures collected

^b^conclusion confirmed by reanalysis conducted as part of project TEENAGE

^c^no analysis of differential effect by original authors, but re-analysed for Project TEENAGE (Lien et al. 2012)

^d^data obtained from authors as unpublished at time of writing

**Table 3:** Quality assessments and methodological parameters for synthesised studies

Study name	Main reference	Study quality	Sample size (school or class/individual)		Retention (numbers analysed) (school or class/individual)		Length of intervention	Length of follow-up
			Intervention	Control	Intervention	Control		
AFLY5	Kipping et al. 2014	Moderate	30/1064	30/1157	30/1024	30/1097	7-11 months	Immediate post-intervention / 1 year
ASSIST	Campbell et al. 2008	Moderate	30/5187	29/5074	30/5058/5044/4966 (post intervention/1 year/2 year)	29/4753/4865/4700	4 months	Immediate post-intervention/ 1 year / 2 years
School fruit scheme	Bere et al. 2010	Weak	27/1488 (total N. Natural experiment - baseline survey prior to adoption or non-adoption)		27/1339 followed up (10 had adopted subscription, 5 free and 12 none)		Unclear	7 years after baseline survey
Crone et al. 2011	Crone et al. 2011	Weak	62/1756	59/1417	62/1010	59/805	2 academic years	Immediate post-intervention / 1 year
EHealth4us	Bannink et al. 2014	Moderate	20/533	20/615	20 / 392	20 / 434	1 month	4 months
			20/554 (2 arms)		20/430			
Energize	Rush et al. 2012	Weak	62	62	62/692	62/660	Variable (at least 18 months)	2 years
ESFA	Ariza et al. 2008	Moderate	16/1083	37/886	13/690	37/603	3 years	Immediate post-intervention
EUDap	Faggiano et al. 2010	Moderate	78 (26/27/25)/1190/1164/1193	65/3532	26/27/24/6 months:1084/1068/1044	64/ 6 months: 3174 18 months: 2730	4 months	6 months/ 18 months
			(basic arm/parent arm/ peer arm)		18 months: 956/972/883			
FatAintPhat	Ezendam et al. 2012	Moderate	11/485	11/145/385/395 (shuttle run/behaviour/anthropometry)	9/398	9/282/333/340	10 weeks	4 months/ 2 years
HEIA	Grydeland et al. 2012	Moderate	12/553	25/975	12/8 months: 541/20 months: 519	25/8 months: 970/20 months: 945	20 months	8 months/ 20 months
KOPS	Plachta-Danielzik 2011	Weak	14/780	32/4217	14/4 years:345/8 years: 239	32/4 years:1419/8 years:950	2-3 weeks	4 years/ 8 years
Avall	Llargues et al. 2011	Weak	8/272	8/237	8/not specified	8/not specified	2 years	Immediate post-intervention
STOPP	Marcus et al.	Moderate	5/1670	5/1465	5/1538	5/1430	1-4 years (mean: 613 days)	Immediate post-intervention
MYTRI	Perry et al. 2009	Moderate	16/6365	16/7698	16/3626	16/4321	2 academic years	Immediate post-intervention
PAS	Koning et al. 2009	Moderate	5/689	4/779	5/5/5/10-22 months: 608/675/588/ 34 months: 603/671/582/ 4 years: 254/291/193	4/10-22 months: 699/ 34 months: 677/ 4 years: 326	1 year	10 months/ 22 months/34 months/ 4 years
			5/771					
			5/698					
			(3 arms)					
Promise	Stallard et al. 2012	Weak	10/1753	9/1604	10/308	9 /242	1 academic year	12 months
			9/1673 (active intervention/attention control) High risk subsample: 392/374	298	9/296 (only high risk followed up)			
PSFBI	Murphy et al. 2011	Moderate	55/2205	56/2145	55/2272	56/2200	12 months	Immediate post-intervention
Smart Lunchbox	Evans et al. 2010	Moderate	44/577	44/671	40/432	43/539	5 months	12 months
SPACE	Toftager et al. 2014	Weak	7/612	7/699	7/515	7/545	2 years	Immediate post- intervention
Pro Children	Te Velde et al. 2008	Moderate	32/990	30/811	32/798	30/674	9 months	Immediate post-intervention

Most interventions with a negative gradient in effect (5 out of 6) focused on diet and physical activity, with 4 reporting widening inequality in obesity. The remaining study with a negative gradient focused on smoking. Studies which reported a positive social gradient were equally distributed (1 study each) among diet and physical activity, diet or physical activity, tobacco and alcohol, tobacco or alcohol.

For interventions based on education alone, 1 reported a negative social gradient in effectiveness, while all others (*N =* 3) reported neutral social gradients. For interventions based on environmental change (without education), 1 reported a positive social gradient in effects, while all others (*N =* 3) reported neutral gradients. For interventions combining education with environmental change and/or family involvement, 5 reported a negative social gradient in effects, 3 a positive social gradient and 4 a neutral gradient. However, multi-level interventions had inconsistent effects on inequality. Studies which demonstrated a positive gradient were all of moderate quality, while half of studies with neutral or negative social gradients were of weak quality.

### Use of process evaluation data to explain or predict impacts on inequality

Of the 20 studies whose authors reported effects on inequality, peer-reviewed publications from a linked process evaluation were available for 7 (although 1 was not available in English). Three process evaluations discussed SES gradients in intervention mechanisms. In all cases, these focused on SES patterning in perceptions of intervention materials. In FatAintPhat [62], pupils in vocational schools rated educational materials more highly and reported being more likely to share them with parents, although pupils in pre-university schools were more likely to fully understand the materials. In Ehealth4US [63], greater appreciation of the educational materials (i.e. rating them as novel and interesting) was reported in lower SES schools. In MYTRI [64], qualitative data indicated that pupils were more engaged in intervention activities in poorer schools. None of these findings of more positive perceptions among lower SES groups translated into bigger effects for these groups.

### Discussion of inequality within intervention studies

Within published articles for the 98 studies, there was little discussion of inequalities in making the case for intervention, or in interpretation of findings. Reports from 19 completed studies (and 3 protocols) referenced socioeconomic inequalities in adolescent health behaviours when defining the problem intervention aimed to address. Twelve studies identified a role for school-based interventions in reaching children from differing socioeconomic backgrounds. Where potential effects on inequalities were discussed, discussion centred primarily on assumptions that universal intervention ensured universal effects. Explicit consideration of mechanisms through which the intervention might affect inequality was limited to 3 studies. Such statements typically reflected assumptions that removing structural barriers and improving access to healthy options was a key means of ensuring that intervention will be accessed by lower SES groups. In interpreting overall outcomes, a small number of authors draw upon assumptions that effects were influenced by SES composition (*N =* 5), most commonly reflecting assumptions that universal interventions work best for poorer groups with most to gain. For example, Lakshman et al. [65] argue that their intervention might have worked better in a deprived sample, stating that “baseline nutritional knowledge was already high and the potential effect size of our intervention could be larger in areas of greater deprivation”.

## Discussion

Effects on inequality have been tested and reported in only one in five peer-reviewed evaluations of universal school-based interventions published since 2008. These analyses indicate that universal school-based interventions have the potential to improve or worsen inequalities, though conclusions regarding how or why are hampered both by a lack of routine testing of effect on inequality, and by biases in consideration of inequality. For example, evaluators of interventions focused on education alone appear least likely to test effects on inequality. Given the often held assumption that educational interventions may make inequality worse, researchers who see reducing SES inequality as a goal of universal intervention perhaps avoid developing or testing interventions based solely on education. Inequality is also rarely considered in studies from North America, though more commonly in European studies.

Within studies which report differential effects, there is inconsistency in how SES is measured, with studies typically providing little justification for their choice of SES measure (e.g. education, SES), or its validity. Furthermore, there is inconsistency in how testing is conducted and reported; some report subgroup effects, some interaction terms and others both. For the most part, where studies do not demonstrate a significant differential effect, test statistics are unreported and it is simply concluded that the intervention was equally effective across SES subgroups. This is despite the fact that studies were usually not powered to detect this interaction, and failure to reach significance is inevitable unless interactions are large [25]. Hence, it is difficult to ascertain whether certain types of intervention are having consistent small to medium effects on inequality which cannot be detected in individual studies. Outcomes are sometimes analysed selectively with, for example, differential effectiveness examined only for variables with a significant main effect, despite the fact that a significant main effect is not a pre-requisite for an effect on inequality.

Nevertheless, the review provides some tentative insights into which types of intervention might affect inequality. No interventions based solely on education reduced inequality, while all interventions which worsened inequality included educational components. By contrast, interventions which resulted in a narrowing of inequality included environmental change components. This offers tentative support for arguments of Mclaren and colleagues [11] and Whitehead [30], that interventions based on structural change may be more likely to narrow inequality. However, effects of interventions which combined education with environmental change were inconsistent. This may indicate differences in the emphases on components at each level, or varying overall effectiveness of components. For example, it is plausible that if an effective education component is combined with an ineffective change in social or physical environments, this may widen inequality.

There was some patterning by behavioural domain. Five studies with a negative social gradient focused on diet and physical activity, with 4 reporting bigger effects on obesity outcomes for the higher SES group, or in one case, a negative effect on obesity in the lower SES group. Notably, alongside smoking interventions, diet and physical activity interventions were the intervention type for which effect on inequality was most commonly tested, perhaps reflecting widespread acknowledgement of the contribution of smoking and obesity to health inequalities. Such studies were more likely to use anthropometric measures of change, rather than relying upon self-reports of behavioural change, which may lack the sensitivity to detect SES differences in effect due to less valid completion in some subgroups [66].

Content analysis of the 98 published evaluations provided key insights into reasons why effects on inequality receive so little attention. Notably, only in a minority of studies was socioeconomic patterning discussed in describing the problem the intervention aimed to address. Hence, SES inequality in health behaviours, despite consistent evidence of its presence throughout adolescence [5], is simply not seen as part of the problem intervention aims to address in most cases. North American studies were least likely to emphasise SES patterning in adolescent health and health behaviours within the rationale for the intervention. This is perhaps linked to high levels of health inequality, and limited political will to address inequalities, in the US compared to many other developed countries [67]. Some European funders such as the NIHR in the UK have begun to mandate analysis of effects on inequality. One study funded by this body stated that they conducted analyses by SES due to research funders’ pressure, though did not report findings in their peer reviewed outcomes paper, due to a lack of statistical power, highlighting a perceived tension between policy need for information on what works for whom vs the desire for scientific purity [25]. Consistent reporting of sub-group effects across intervention studies, even where individual studies are underpowered, would allow systematic reviewers and meta-analysts to pool effects across studies to identify whether there are consistent interaction across studies.

School-based interventions were sometimes portrayed as inevitably reaching all SES groups and having universal effect; an assumption challenged by the finding that many universal interventions worsened or narrowed inequality. A more minority assumption, contradicted by the finding of variable effects of school-based intervention on inequality was that interventions would inevitably work better for groups who have the most to gain, due to ceiling effects in more affluent groups. Theory or empirical evidence on effects on inequality were rarely drawn upon throughout evaluations. A minority of studies included process evaluations, some of which emphasised SES differences in perceptions of the intervention, none of which translated into greater effects for lower SES groups. None provided more in-depth insights into how the contexts in which lower or higher SES pupils experienced the intervention impacted its effectiveness.

A strength of this review is the fact that the search strategy incorporated seven databases, ensuring a high level of coverage. However, this could have been extended to grey literature and research published in languages other than English. The review, in part for reasons of resource, also covers a relatively narrow time period, and earlier evaluations may have contributed different understandings of intervention effects on inequality.

## Conclusions

Nevertheless, the review indicates that universal school-based interventions may narrow inequalities, or make them worse. Peer-reviewed reports of evaluation studies indicate that SES inequalities are rarely considered as a part of the problem intervention stakeholders are aiming to address, while assessment of impact on inequality remains a minority activity. The review provides support for notions that interventions based on education may be more likely to widen inequality, with those based on environmental change more likely to narrow it. However, conclusions are highly tentative due to the fact that the vast majority of relevant studies provide no such analysis, while those that do tend to be linked to specific locations and types of intervention. At present, such analyses are almost exclusive to European studies; much could be learned through more routine assessment of inequalities impact in other contexts, including North America, which currently generates a large proportion of intervention studies, though pays least attention to effect on inequality. There is also a need for guidance on the most appropriate methods for analysis and reporting of sub-group effects in order to allow for comparison across studies. Authors of evaluations need to clearly justify the measure of SES used. Different dimensions of SES may function differently in shaping health across contexts – in the US, for example, the racialization of inequality may mean that ethnicity in itself can be considered a valid proxy measure of SES in that context. Hence, while it may not be appropriate to standardise SES measures across all studies, the measure selected should be justified with reference to its role in shaping the health outcomes under investigation in the context where the intervention is delivered. It is vital that authors present interaction effects and sub-group analyses fully, regardless of whether the individual study was sufficiently powered to detect a moderating effect. Accumulation of consistently applied methods to assess intervention effects on inequality could allow future research to use methods such as meta-regression or qualitative comparative analysis to examine what features of interventions were associated with positive, negative or neutral effects on health inequalities, in what contexts. To facilitate this, guidance for the conduct, analysis and reporting of evaluations of complex interventions should include recommendations on measuring, analysing and reporting effects on inequality. Greater use of process evaluation to facilitate understandings of how interventions are experienced by young people with varying socioeconomic backgrounds may help in building theory and providing deeper insights into why some school-based interventions worsen inequalities while others reduce it.

## Availability of data and materials 

Not applicable.

## Additional files

Additional file 1: Full list of studies included (including those which did and dod not examine differential effectsby SES). (RTF 1427 kb) Additional file 2: Search strategy. (DOCX 18 kb) Additional file 3: Prisma checklist. (PDF 248 kb)
