# Supplementary material for: Socioeconomic gradients in the effects of universal school-based health behaviour interventions: a systematic review of intervention studies
Source: BMC Public Health. 2015 Sep 17;15:907. doi: 10.1186/s12889-015-2244-x (PMC4574356; doi:10.1186/s12889-015-2244-x)
Supplement: Additional file 1: — Full list of studies included (including those which did and dod not examine differential effectsby SES). (RTF 1427 kb) [file 12889_2015_2244_MOESM1_ESM.rtf]

References to all 98 studies included (prior to extraction of differential effects data)
Andersen, R., Biltoft-Jensen, A., Christensen, T., Andersen, E. W., Ege, M., Thorsen, A. V., et al. (2014). Dietary effects of introducing school meals based on the New Nordic Diet–a randomised controlled trial in Danish children. The OPUS School Meal Study. British Journal of Nutrition, 111(11), 1967-1976.
Andrews, J. A., Gordon, J. S., Hampson, S. E., Christiansen, S. M., Gunn, B., Slovic, P., et al. (2011). Short-Term Efficacy of Click City: Tobacco: Changing Etiological Mechanisms Related to the Onset of Tobacco Use. Prevention Science, 12(1), 2011-2102.
Angelopoulos, P. D., Milionis, H. J., Grammatikaki, E., Moschonis, G., & Manios, Y. (2009). Changes in BMI and blood pressure after a school based intervention: The CHILDREN study. European Journal of Public Health, 19(3), June-325.
Ariza, C., Nebot, M., Tomas, Z., Gimenez, E., Valmayor, S., Tarilonte, V., et al. (2008). Longitudinal effects of the European smoking prevention framework approach (ESFA) project in Spanish adolescents. European Journal of Public Health, 18(5), October-497.
Balvig, F., & Holmberg, L. (2011). The ripple effect: A randomized trial of a social norms intervention in a Danish middle school setting. [References]. Journal of Scandinavian Studies in Criminology and Crime Prevention(1), 3-19.
Bannink, R., Joosten-van, Z. E., Looij-Jansen, P., van, A. E., & Raat, H. (2012). Evaluation of computer-tailored health education ('E-health4Uth') combined with personal counselling ('E-health4Uth + counselling') on adolescents' behaviours and mental health status: design of a three-armed cluster randomised controlled trial. BMC Public Health, 12(pp 1083), 2012.
Bere, E., Hilsen, M., & Klepp, K. I. (2010). Effect of the nationwide free school fruit scheme in Norway. British Journal of Nutrition, 104(4), 589-594.
Bodin, M. C., & Strandberg, A. K. (2011). The Orebro prevention programme revisited: a cluster-randomized effectiveness trial of programme effects on youth drinking. Addiction (Abingdon, England), 106(12), Dec-2143.
Bonsergent, E., Agrinier, N., Thilly, N., Tessier, S., Legrand, K., Lecomte, E., et al. (2013). Overweight and obesity prevention for adolescents: A cluster randomized controlled trial in a school setting. American Journal of Preventive Medicine, 44(1), January-39.
Boyle-Holmes, T., Grost, L., Russell, L., Laris, B. A., Robin, L., Haller, E., et al. (2010). Promoting elementary physical education: results of a school-based evaluation study. Health Education & Behavior, 37(3), 377-389.
Buller, D. B., Borland, R., Woodall, W. G., Hall, J. R., Hines, J. M., Burris-Woodall, P., et al. (2008). Randomized trials on consider this, a tailored, internet-delivered smoking prevention program for adolescents. Health Education and Behavior, 35(2), April-281.
Campbell, R., Starkey, F., Holliday, J., Audrey, S., Bloor, M., Parry-Langdon, N., et al. (2008). An informal school-based peer-led intervention for smoking prevention in adolescence (ASSIST): a cluster randomised trial. The Lancet, 371(9624), 20080510/20080516-20080511602.
Chatzisarantis, N. L. D., & Hagger, M. S. (2009). Effects of an intervention based on self-determination theory on self-reported leisure-time physical activity participation. Psychology and Health, 24(1), January-48.
Cradock, A. L., McHugh, A., Mont-Ferguson, H., Grant, L., Barrett, J. L., Wang, Y. C., et al. (2011). Effect of school district policy change on consumption of sugar-sweetened beverages among high school students, Boston, Massachusetts, 2004-2006. Preventing Chronic Disease, 8(4), Jul.
Crone, M. R., Spruijt, R., Dijkstra, N. S., Willemsen, M. C., & Paulussen, T. G. W. M. (2011). Does a smoking prevention program in elementary schools prepare children for secondary school? Preventive Medicine, 52(1), January-59.
Cunha, D. B., Souza, B., Pereira, R. A., & Sichieri, R. (2013). Effectiveness of a Randomized School-Based Intervention Involving Families and Teachers to Prevent Excessive Weight Gain among Adolescents in Brazil. PLoS ONE, 8(2), e57498.
D'Amico, E. J., Tucker, J. S., Miles, J. N. V., Zhou, A. J., Shih, R. A., & Green, Jr. (2012). Preventing Alcohol Use with a Voluntary After-School Program for Middle School Students: Results from a Cluster Randomized Controlled Trial of CHOICE. Prevention Science, 13(4), August-425.
Donnelly, J. E., Greene, J. L., Gibson, C. A., Smith, B. K., Washburn, R. A., Sullivan, D. K., et al. (2009). Physical Activity Across the Curriculum (PAAC): A randomized controlled trial to promote physical activity and diminish overweight and obesity in elementary school children. [References]. Preventive Medicine: An International Journal Devoted to Practice and Theory(4), 336-341.
Donnelly, J. E., Greene, J. L., Gibson, C. A., Sullivan, D. K., Hansen, D. M., Hillman, C. H., et al. (2013). Physical activity and academic achievement across the curriculum (A + PAAC): rationale and design of a 3-year, cluster-randomized trial. BMC Public Health, 13(pp 307), 2013.
Dreyhaupt, J., Koch, B., Wirt, T., Schreiber, A., Brandstetter, S., Kesztyus, D., et al. (2012). Evaluation of a health promotion program in children: Study protocol and design of the cluster-randomized Baden-Wurttemberg primary school study [DRKS-ID: DRKS00000494]. BMC Public Health, 12(pp 157), 2012.
Dunn, L. L., Venturanza, J. A., Walsh, R. J., & Nonas, C. A. (2012). An observational evaluation of move-to-improve, a classroom-based physical activity program, New York City schools, 2010. Preventing Chronic Disease, 9(pp E146), 2012.
Dzewaltowski, D. A., Estabrooks, P. A., Welk, G., Hill, J., Milliken, G., Karteroliotis, K., et al. (2009). Healthy youth places: A randomized controlled trial to determine the effectiveness of facilitating adult and youth leaders to promote physical activity and fruit and vegetable consumption in middle schools. Health Education and Behavior, 36(3), June-600.
Elinder, L. S., Heinemans, N., Hagberg, J., Quetel, A. K., & Hagstromer, M. (2012). A participatory and capacity-building approach to healthy eating and physical activity - SCIP-school: A 2-year controlled trial. International Journal of Behavioral Nutrition and Physical Activity, 9.
Emamhadi, M. A., Khodabandeh, F., Jalilvand, M., Hadian, M., & Heydari, G. R. (2012). Efficacy of different methods in decreasing the students' tendency towards smoking. [References]. International Journal of Child and Adolescent Health(1), 47-51.
Engelen, L., Bundy, A. C., Naughton, G., Simpson, J. M., Bauman, A., Ragen, J., et al. (2013). Increasing physical activity in young primary school children - it's child's play: A cluster randomised controlled trial. Preventive Medicine, 56(5), May-325.
Evans, C. E. L., Greenwood, D. C., Thomas, J. D., Cleghorn, C. L., Kitchen, M. S., & Cade, J. E. (2010). SMART lunch box intervention to improve the food and nutrient content of children's packed lunches: UK wide cluster randomised controlled trial. Journal of Epidemiology and Community Health, 64(11), November-976.
Ezendam, N. P. M., Brug, J., & Oenema, A. (2012). Evaluation of the web-based computer-tailored FATaintPHAT intervention to promote energy balance among adolescents: Results from a school cluster randomized trial. Archives of Pediatrics and Adolescent Medicine, 166(3), March-255.
Faggiano, F., Galanti, M. R., Bohrn, K., Burkhart, G., Vigna-Taglianti, F., Cuomo, L., et al. (2008). The effectiveness of a school-based substance abuse prevention program: EU-Dap cluster randomised controlled trial. Preventive Medicine, 47(5), November-543.
Gabrhelik, R., Duncan, A., Miovsky, M., Furr-Holden, C. D. M., Stastna, L., & Jurystova, L. (2012). "Unplugged": A school-based randomized control trial to prevent and reduce adolescent substance use in the Czech Republic. Drug and Alcohol Dependence, 124(1-2), 01-87.
Gentile, D. A., Welk, G., Eisenmann, J. C., Reimer, R. A., Walsh, D. A., Russell, D. W., et al. (2009). Evaluation of a multiple ecological level child obesity prevention program: Switch what you Do, View, and Chew. BMC medicine, 7(pp 49), 2009.
Giles, C. M., Kenney, E. L., Gortmaker, S. L., Lee, R. M., Thayer, J. C., Mont-Ferguson, H., et al. (2012). Increasing water availability during afterschool snack: Evidence, strategies, and partnerships from a group randomized trial. American Journal of Preventive Medicine, 43(3 SUPPL.2), September-S142.
Gomes de Barros, M. V., Nahas, M. V., Hallal, P. C., Cazuzade Farias, J., Jr., Florindo, A. A., & Honda de Barros, S. S. (2009). Effectiveness of a school-based intervention on physical activity for high school students in Brazil: The Saude na Boa project. [References]. Journal of physical activity & health(2), 163-169.
Gorini, G., Carreras, G., Bosi, S., Tamelli, M., Monti, C., Storani, S., et al. (2014). Effectiveness of a school-based multi-component smoking prevention intervention: The LdP cluster randomized controlled trial. Preventive Medicine, 61, 6-13.
Grydeland, M., Bergh, I. H., Bjelland, M., Lien, N., Andersen, L. F., Ommundsen, Y., et al. (2013). Intervention effects on physical activity: The HEIA study - a cluster randomized controlled trial. International Journal of Behavioral Nutrition and Physical Activity, 10.
Hallgren, M. A., SjApglund, T., KallmAcon, H. A. Y., & AndrAcoasson, S. (2011). Modifying alcohol consumption among high school students. Health Education, 111(3), 216-229.
Hands, B., Larkin, D., Rose, E., Parker, H., & Smith, A. (2011). Can young children make active choices? Outcomes of a feasibility trial in seven-year-old children. [References]. Early Child Development and Care(5), 625-637.
Hatzis, C. M., Papandreou, C., & Kafatos, A. G. (2010). School health education programs in Crete: Evaluation of behavioural and health indices a decade after initiation. Preventive Medicine, 51(3-4), September-267.
Hawkins, J. D., Oesterle, S., Brown, E. C., Arthur, M. W., Abbott, R. D., Fagan, A. A., et al. (2009). Results of a type 2 translational research trial to prevent adolescent drug use and delinquency: A test of communities that care. Archives of Pediatrics and Adolescent Medicine, 163(9), September-798.
He, F. J., Wu, Y., Ma, J., Feng, X., Wang, H., Zhang, J., et al. (2013). A School-based Education Programme to Reduce salt intake in children and their families (School-EduSalt): Protocol of a cluster randomised controlled trial. BMJ Open, 3(7), e003388.
He, M., Beynon, C., Sangster, B. M., St, O. R., Stewart, S., Khoshaba, L., et al. (2009). Impact evaluation of the Northern Fruit and Vegetable Pilot Programme - a cluster-randomised controlled trial. Public Health Nutrition, 12(11), Nov-2208.
Hoppu, U., Lehtisalo, J., Kujala, J., Keso, T., Garam, S., Tapanainen, H., et al. (2010). The diet of adolescents can be improved by school intervention. Public Health Nutrition, 13(6 A), Jun-979.
Hu, X., Zhang, Q., Liu, A., Fang, H., Hao, L., Duan, Y., et al. (2010). The nutrition-based comprehensive intervention study on childhood obesity in China (NISCOC): a randomised cluster controlled trial. BMC Public Health, 10(pp 229), 2010.
Isensee, B., Hansen, J., Maruska, K., & Hanewinkel, R. (2014). Effects of a school-based prevention programme on smoking in early adolescence: A 6-month follow-up of the 'Eigenstandig werden' cluster randomised trial. BMJ Open, 4(1), e004422.
Jago, R., Sebire, S. J., Davies, B., Wood, L., Edwards, M. J., Banfield, K., et al. (2014). Randomised feasibility trial of a teaching assistant led extracurricular physical activity intervention for 9 to 11 year olds: Action 3: 30. International Journal of Behavioral Nutrition and Physical Activity, 11(1), 114.
Johnson, C. C., Myers, L., Webber, L. S., Boris, N. W., He, H., & Brewer, D. (2009). A school-based environmental intervention to reduce smoking among high school students: the Acadiana Coalition of Teens against Tobacco (ACTT). International Journal of Environmental Research & Public Health [Electronic Resource], 6(4), 1298-1316.
Keyte, J., Harris, S., Margetts, B., Robinson, S., & Baird, J. (2012). Engagement with the National Healthy Schools Programme is associated with higher fruit and vegetable consumption in primary school children. Journal of human nutrition and dietetics : the official journal of the British Dietetic Association, 25(2), Apr-160.
Kipping, R. R., Howe, L. D., Jago, R., Campbell, R., Wells, S., Chittleborough, C. R., et al. (2014). Effect of intervention aimed at increasing physical activity, reducing sedentary behaviour, and increasing fruit and vegetable consumption in children: Active for Life Year 5 (AFLY5) school based cluster randomised controlled trial. BMJ, 348, g3256.
Kocken, P. L., Eeuwijk, J., Van Kesteren, N. M., Dusseldorp, E., Buijs, G., Bassa-Dafesh, Z., et al. (2012). Promoting the Purchase of Low-Calorie Foods From School Vending Machines: A Cluster-Randomized Controlled Study. Journal of School Health, 82(3), March-122.
Koning, I. M., van den Eijnden, R. J., Verdurmen, J. E., Engels, R. C., & Vollebergh, W. A. (2011). Long-term effects of a parent and student intervention on alcohol use in adolescents: A cluster randomized controlled trial. American Journal of Preventive Medicine, 40(5), May-547.
Kremer, P., Waqa, G., Vanualailai, N., Schultz, J. T., Roberts, G., Moodie, M., et al. (2011). Reducing unhealthy weight gain in Fijian adolescents: Results of the Healthy Youth Healthy Communities study. Obesity Reviews, 12(SUPPL.#2), November.
Kriemler, S., Zahner, L., Schindler, C., Meyer, U., Hartmann, T., Hebestreit, H., et al. (2010). Effect of school based physical activity programme (KISS) on fitness and adiposity in primary schoolchildren: cluster randomised controlled trial. BMJ (Clinical research ed.), 340(pp c785), 2010.
Krolner, R., Suldrup, J. T., Aarestrup, A. K., Hjollund, C. A., Christensen, A. M., & Due, P. (2012). The Boost study: design of a school- and community-based randomised trial to promote fruit and vegetable consumption among teenagers. BMC Public Health, 12(pp 191), 2012.
La, T. G., Chiaradia, G., Monte, L., Moretti, C., Mannocci, A., Capitanio, D., et al. (2010). A randomised controlled trial of a school-based intervention to prevent tobacco use among children and adolescents in Italy. Journal of Public Health, 18(6), December-542.
Lakshman, R. R., Sharp, S. J., Ong, K. K., & Forouhi, N. G. (2010). A novel school-based intervention to improve nutrition knowledge in children: cluster randomised controlled trial. BMC Public Health, 10(pp 123), 2010.
Llargues, E., Franco, R., Recasens, A., Nadal, A., Vila, M., Perez, M. J., et al. (2011). Assessment of a school-based intervention in eating habits and physical activity in school children: The AVall study. Journal of Epidemiology and Community Health, 65(10), October-901.
Lonsdale, C., Rosenkranz, R. R., Sanders, T., Peralta, L. R., Bennie, A., Jackson, B., et al. (2013). A cluster randomized controlled trial of strategies to increase adolescents' physical activity and motivation in physical education: Results of the Motivating Active Learning in Physical Education (MALP) trial. Preventive Medicine, 57(5), November-702.
Lotrean, L. M., Dijk, F., Mesters, I., Ionut, C., & De, V. H. (2010). Evaluation of a peer-led smoking prevention programme for Romanian adolescents. Health Education Research, 25(5), Oct-814.
Luna-Adame, M., Carrasco-Gimenez, T. J., & del Mar Rueda-Garcia, M. (2013). Evaluation of the effectiveness of a smoking prevention program based on the 'Life Skills Training' approach. [References]. Health Education Research(4), 673-682.
Malmberg, M., Overbeek, G., Kleinjan, M., Vermulst, A., Monshouwer, K., Lammers, J., et al. (2010). Effectiveness of the universal prevention program 'Healthy School and Drugs': study protocol of a randomized clustered trial. BMC Public Health, 10(pp 541), 2010.
Marcus, C., Nyberg, G., Nordenfelt, A., Karpmyr, M., Kowalski, J., & Ekelund, U. (2009). A 4-year, cluster-randomized, controlled childhood obesity prevention study: STOPP. International Journal of Obesity, 33(4), April-417.
Martinez-Vizcaino, V., Sanchez-Lopez, M., Salcedo-Aguilar, F., Notario-Pacheco, B., Solera-Martinez, M., Moya-Martinez, P., et al. (2012). Protocol of a randomized cluster trial to assess the effectiveness of the MOVI-2 program on overweight prevention in schoolchildren. Revista Espanola de Cardiologia, 65(5), 427-433.
McKay, M. T., McBride, N. T., Sumnall, H. R., & Cole, J. C. (2012). Reducing the harm from adolescent alcohol consumption: results from an adapted version of SHAHRP in Northern Ireland. Journal of Substance Use, 17(2), 98-121.
Midford, R., Mitchell, J., Lester, L., Cahill, H., Foxcroft, D., Ramsden, R., et al. (2014). Preventing alcohol harm: Early results from a cluster randomised, controlled trial in Victoria, Australia of comprehensive harm minimisation school drug education. International Journal of Drug Policy, 25(1), January.
Morgenstern, M., Wiborg, G., Isensee, B., & Hanewinkel, R. (2009). School-based alcohol education: Results of a cluster-randomized controlled trial. Addiction, 104(3), March-412.
Murphy, S., Moore, G. F., Tapper, K., Lynch, R., Clarke, R., Raisanen, L., et al. (2011). Free healthy breakfasts in primary schools: a cluster randomised controlled trial of a policy intervention in Wales, UK. Public Health Nutrition, 14(2), Feb-226.
O'Neill, J. M., Clark, J. K., & Jones, J. A. (2011). Promoting mental health and preventing substance abuse and violence in elementary students: A randomized control study of the michigan model for health. Journal of School Health, 81(6), June-330.
Perry, C. L., Stigler, M. H., Arora, M., & Reddy, K. S. (2009). Preventing tobacco use among young people in India: Project MYTRI. American Journal of Public Health, 99(5), 01-906.
Plachta-Danielzik, S., Landsberg, B., Lange, D., Seiberl, J., & Muller, M. J. (2011). Eight-year follow-up of school-based intervention on childhood overweight - The Kiel obesity prevention study. Obesity Facts, 4(1), February-43.
Reinaerts, E., Crutzen, R., Candel, M., De Vries, N. K., & De, N. J. (2008). Increasing fruit and vegetable intake among children: Comparing long-term effects of a free distribution and a multicomponent program. Health Education Research, 23(6), December-996.
Ringwalt, C. L., Clark, H. K., Hanley, S., Shamblen, S. R., & Flewelling, R. L. (2010). The effects of project ALERT one year past curriculum completion. Prevention Science, 11(2), 2010-2184.
Rohrbach, L. A., Sun, P., & Sussman, S. (2010). One-year follow-up evaluation of the Project Towards No Drug Abuse (TND) dissemination trial. [References]. Preventive Medicine: An International Journal Devoted to Practice and Theory(3-4), 313-319.
Rush, E., Reed, P., McLennan, S., Coppinger, T., Simmons, D., & Graham, D. (2012). A school-based obesity control programme: Project Energize. Two-year outcomes. British Journal of Nutrition, 107(4), 28-587.
Sacchetti, R., Ceciliani, A., Garulli, A., Dallolio, L., Beltrami, P., & Leoni, E. (2013). Effects of a 2-Year School-Based Intervention of Enhanced Physical Education in the Primary School. Journal of School Health, 83(9), 639-646.
Santos, R. G., Durksen, A., Rabbanni, R., Chanoine, J. P., Miln, A. L., Mayer, T., et al. (2014). Effectiveness of peer-based healthy living lesson plans on anthropometric measures and physical activity in elementary school students a cluster randomized trial. JAMA Pediatrics, 168(4), April.
Shamah, L. T., Morales, R. C., Amaya, C. C., Salazar, C. A., Jimenez, A. A., & Mendez, G. H. I. (2012). Effectiveness of a diet and physical activity promotion strategy on the prevention of obesity in Mexican school children. BMC Public Health, 12(pp 152), 2012.
Sichieri, R., Paula, T. A., de Souza, R. A., & Veiga, G. V. (2009). School randomised trial on prevention of excessive weight gain by discouraging students from drinking sodas. Public Health Nutrition, 12(2), Feb-202.
Siegrist, M., Hanssen, H., Lammel, C., Haller, B., & Halle, M. (2011). A cluster randomised school-based lifestyle intervention programme for the prevention of childhood obesity and related early cardiovascular disease (JuvenTUM 3). BMC Public Health, 11(pp 258), 2011.
Sloboda, Z., Stephens, R. C., Stephens, P. C., Grey, S. F., Teasdale, B., Hawthorne, R. D., et al. (2009). The Adolescent Substance Abuse Prevention Study: A randomized field trial of a universal substance abuse prevention program. Drug and Alcohol Dependence, 102(1-3), 01-10.
Stallard, P., Montgomery, A. A., Araya, R., Anderson, R., Lewis, G., Sayal, K., et al. (2010). Protocol for a randomised controlled trial of a school based cognitive behaviour therapy (CBT) intervention to prevent depression in high risk adolescents (PROMISE). Trials, 11.
Stamm-Balderjahn, S., Groneberg, D. A., Kusma, B., Jagota, A., & Schonfeld, N. (2012). Smoking prevention in school students. [References]. Deutsches Arzteblatt International(44), 746-752.
Suchert, V., Isensee, B., Hansen, J., Johannsen, M., Krieger, C., Muller, K., et al. (2013). " lauft." - a school-based multi-component program to establish a physically active lifestyle in adolescence: Study protocol for a cluster-randomized controlled trial. Trials, 14(1), 416.
Tarro, L., Llauradó, E., Albaladejo, R., Moriña, D., Arija, V., Solà, R., et al. (2014). A primary-school-based study to reduce the prevalence of childhood obesity—the EdAl (Educació en Alimentació) study: a randomized controlled trial. Trials, 15(1), 58.
Te Velde, S. J., Brug, J., Wind, M., Hildonen, C., Bjelland, M., Perez-Rodrigo, C., et al. (2008). Effects of a comprehensive fruit- and vegetable-promoting school-based intervention in three European countries: The Pro Children Study. British Journal of Nutrition, 99(4), April-903.
Toftager, M., Christiansen, L. B., Ersbøll, A. K., Kristensen, P. L., Due, P., & Troelsen, J. (2014). Intervention Effects on Adolescent Physical Activity in the Multicomponent SPACE Study: A Cluster Randomized Controlled Trial. PLoS ONE, 9(6), e99369.
Toumbourou, J. W., Gregg, M. E. D., Shortt, A. L., Hutchinson, D. M., & Slaviero, T. M. (2013). Reduction of adolescent alcohol use through family-school intervention: A Randomized trial. Journal of Adolescent Health, 53(6), December-784.
Van Lier, P. A. C., Huizink, A., & Crijnen, A. (2009). Impact of a preventive intervention targeting childhood disruptive behavior problems on tobacco and alcohol initiation from age 10 to 13 years. Drug and Alcohol Dependence, 100(3), 01-233.
Vereecken, C., Huybrechts, I., van, H. H., Martens, V., Wittebroodt, I., & Maes, L. (2009). Results from a dietary intervention study in preschools "Beastly Healthy at School". International Journal of Public Health, 54(3), 142-149.
Verloigne, M., Bere, E., Van, L. W., Maes, L., Lien, N., Vik, F. N., et al. (2012). The effect of the UP4FUN pilot intervention on objectively measured sedentary time and physical activity in 10-12 year old children in Belgium: the ENERGY-project. BMC Public Health, 12(pp 805), 2012.
Viggiano, E., Viggiano, A., Vicidomini, C., Di, C. A., Andreozzi, E., Romano, V., et al. (2012). Kaledo, a new educational board-game for nutrition education: Cluster randomized trial of healthy lifestyle promotion. Obesity Facts.Conference: 19th European Congress on Obesity, ECO2012 Lyon France.Conference Start: 20120509 Conference End: 20120512.Conference Publication: (var.pagings), 5(pp 260), May.
Vincus, A. A., Ringwalt, C., Harris, M. S., & Shamblen, S. R. (2010). A short-term, quasi-experimental evaluation of dare's revised elementary school curriculum. Journal of drug education, 40(1), 37-49.
Vitoria, P. D., Silva, S. A., & Vries, H. D. (2011). Longitudinal evaluation of a smoking prevention program for adolescents. Revista de Saude Publica, 45(2), Apr-354.
Wen, L. M., Fry, D., Merom, D., Rissel, C., Dirkis, H., & Balafas, A. (2008). Increasing active travel to school: Are we on the right track? A cluster randomised controlled trial from Sydney, Australia. Preventive Medicine, 47(6), December-618.
Wenzel, V., Weichold, K., & Silbereisen, R. K. (2009). The life skills program IPSY: Positive influences on school bonding and prevention of substance misuse. Journal of Adolescence, 32(6), December-1401.
West, B., Abatemarco, D., Ohman-Strickland, P. A., Zec, V., Russo, A., & Milic, R. (2008). Project Northland in Croatia: results and lessons learned. Journal of drug education, 38(1), 2008-2070.
Wilson, D. B., Jones, R. M., McClish, D., Westerberg, A. L., & Danish, S. (2012). Fruit and vegetable intake among rural youth following a school-based randomized controlled trial. Preventive Medicine, 54(2), 01-156.
Wyatt, K. M., Lloyd, J. J., Abraham, C., Creanor, S., Dean, S., Densham, E., et al. (2013). The Healthy Lifestyles Programme (HeLP), a novel school-based intervention to prevent obesity in school children: study protocol for a randomised controlled trial. Trials [Electronic Resource], 14, 95.
Yýldýrým, M., Arundell, L., Cerin, E., Carson, V., Brown, H., Crawford, D., et al. (2013). What helps children to move more at school recess and lunchtime? Mid-intervention results from Transform-Us! cluster-randomised controlled trial. British journal of sports medicine, bjsports-2013-092466.
Yin, Z., Moore, J. B., Johnson, M. H., Vernon, M. M., & Gutin, B. (2012). The impact of a 3-year after-school obesity prevention program in elementary school children. Childhood Obesity, 8(1), 60-70.

Reference	Study name	Country	Behaviour type	
(Donnelly, Greene et al. 2013)	Physical Activity and Academic Achievement across the Curriculum  (A+PAAC)	United states	Physical activity	
(Jago, Sebire et al. 2014)	Action 3:30	United Kingdom	Physical activity	
(Johnson, Myers et al. 2009)	ACTT	United States	Smoking	
(Kipping, Howe et al. 2014)	AFLY5	United Kingdom	Physical activity, fruit and vegetable intake	
(Ringwalt, Clark et al. 2010)	ALERT	United States	Cigarettes, alcohol	
(Sloboda, Stephens et al. 2009)	ASAPS	United States	Smoking, alcohol	
(Campbell, Starkey et al. 2008)	ASSIST	United Kingdom	Smoking	
(Llargues, Franco et al. 2011)	AVall	Spain	Physical activity, diet	
(Dreyhaupt, Koch et al. 2012)	Baden-Württemberg primary
school study	Germany	Physical activity, diet	
(Vereecken, Huybrechts et al. 2009)	Beastly Healthy	Belgium	Diet	
(Krolner, Suldrup et al. 2012)	BOOST	Denmark	Fruit and vegetable intake	
(Chatzisarantis and Hagger 2009)	Chatzisarantis	United Kingdom	Physical activity	
(Engelen, Bundy et al. 2013)	Child's Play	Australia	Physical activity	
(Angelopoulos, Milionis et al. 2009)	Children	Greece	Physical activity, diet	
(D'Amico, Tucker et al. 2012)	Choice	United States	Alcohol	
(Andrews, Gordon et al. 2011)	Click City	United States	Smoking	
(Hawkins, Oesterle et al. 2009)	Communities That Care	United States	Smoking, alcohol	
(Buller, Borland et al. 2008)	Consider This	Australia and United States	Smoking	
(Cradock, McHugh et al. 2011)	Cradock	United States	Sugar sweetened beverage consumption	
(Crone, Spruijt et al. 2011)	Crone	Netherlands	Smoking	
(Cunha, Souza et al. 2013)	Cunha	Brazil	Diet	
(Midford, Mitchell et al. 2014)	DEVS	Australia	Alcohol	
(Tarro, Llauradó et al. 2014)	EDUCenALIM	Spain	Physical activity, diet	
(He, Wu et al. 2013)	EduSalt	China	Salt intake	
(Bannink, Joosten-van et al. 2012)	EHealth4US	Netherlands	Smoking, alcohol	
(Isensee, Hansen et al. 2014)	Eigenständig werden	Germany	Smoking	
(Emamhadi, Khodabandeh et al. 2012)	Emam-Hadi	Iran	Smoking	
(Rush, Reed et al. 2012)	Project Energize	New Zealand	Physical activity, diet	
(Verloigne, Bere et al. 2012)	ENERGY	Belgium	Physical activity	
(Boyle-Holmes, Grost et al. 2010)	Michigan's Exemplary Physical Education Curriculum
(EPEC)	United States	Physical activity	
(Ariza, Nebot et al. 2008)	European Smoking Prevention Framework  (ESFA)	Spain	Smoking	
(Faggiano, Galanti et al. 2008)	EU-Dap	Seven European countries	Smoking, alcohol	
(Reinaerts, Crutzen et al. 2008)	F&V	Netherlands	Fruit and vegetable consumption	
(Ezendam, Brug et al. 2012)	FATaintPHAT	Netherlands	Physical activity, diet	
(Hoppu, Lehtisalo et al. 2010)	FINLAND	Finland	Diet	
(Bere, Hilsen et al. 2010)	Free School Fruit Scheme	Norway	Diet	
(Yin, Moore et al. 2012)	Georgia FitKid	United States	Physical activity	
(Hatzis, Papandreou et al. 2010)	Hatzis	Greece (Crete)	Physical activity, diet, smoking	
(Santos, Durksen et al. 2014)	Healthy Buddies	Canada	Physical activity, diet	
(Malmberg, Overbeek et al. 2010)	Healthy School and Drugs	Netherlands	Smoking, alcohol	
(Grydeland, Bergh et al. 2013)	HEIA	Norway	Physical activity, diet	
(Kremer, Waqa et al. 2011)	Healthy Youth Healthy Communities	Fiji	Physical activity, diet	
(Dzewaltowski, Estabrooks et al. 2009)	Healthy Youth Places	United States	Physical activity, fruit and vegetable consumption	
(Wyatt, Lloyd et al. 2013)	HeLP	United Kingdom	Physical activity, diet	
(Wenzel, Weichold et al. 2009)	IPSY	Germany	Alcohol	
(Siegrist, Hanssen et al. 2011)	JuvenTUM 3	Germany	Physical activity, diet	
(Viggiano, Viggiano et al. 2012)	Kaledo	Italy	Diet	
(Kriemler, Zahner et al. 2010)	KISS	Switzerland	Physical activity	
(Kocken, Eeuwijk et al. 2012)	Kocken	Netherlands	Diet	
(Plachta-Danielzik, Landsberg et al. 2011)	KOPS	Germany	Physical activity, diet	
(Lakshman, Sharp et al. 2010)	Lakshman	United Kingdom	Diet	
(La, Chiaradia et al. 2010)	La Torre	Italy	Smoking	
(Suchert, Isensee et al. 2013)	läuft	Germany	Physical activity	
(Gorini, Carreras et al. 2014)	LdP	Italy	Smoking	
(Wilson, Jones et al. 2012)	Lift+	United States	Fruit and vegetable intake	
(Lotrean, Dijk et al. 2010)	Lotrean	Romania	Smoking	
(Luna-Adame, Carrasco-Gimenez et al. 2013)	Luna-Adame	Spain	Smoking	
(Lonsdale, Rosenkranz et al. 2013)	MALP	Australia	Physical activity	
(O'Neill, Clark et al. 2011)	Michigan Model for Health	United States	Smoking, alcohol	
(Morgenstern, Wiborg et al. 2009)	Morganstern	Germany	Alcohol	
(Dunn, Venturanza et al. 2012)	Move-To-Improve	United States	Physical activity	
(Martinez-Vizcaino, Sanchez-Lopez et al. 2012)	MOVI-2	Spain	Physical activity	
(Perry, Stigler et al. 2009)	MYTRI	India	Smoking	
(Keyte, Harris et al. 2012)	National Healthy Schools Programme	United Kingdom	Fruit and vegetable consumption	
(Hu, Zhang et al. 2010)	NISCOC	China	Physical activity, diet	
(He, Beynon et al. 2009)	Northern Fruit and Vegetable	Canada	Fruit and vegetable consumption	
(Shamah, Morales et al. 2012)	Nutrition on the go	Mexico	Physical activity, diet	
(Andersen, Biltoft-Jensen et al. 2014)	OPUS	Denmark	Diet	
(Bodin and Strandberg 2011)	Örebro	Sweden	Diet	
(Donnelly, Greene et al. 2009)	PAAC	United States	Physical activity	
(Koning, van den Eijnden et al. 2011)	PAS	Netherlands	Alcohol	
(Bonsergent, Agrinier et al. 2013)	PRALIMAP	France	Physical activity, diet	
(Hallgren, SjApglund et al. 2011)	PRIME for Life	Sweden	Alcohol	
(Te Velde, Brug et al. 2008)	Pro Children	Spain, Norway and Netherlands	Fruit and vegetable intake 	
(Stallard, Montgomery et al. 2010)	PROMISE	United Kingdom	Alcohol	
(Murphy, Moore et al. 2011)	Primary School Free School Breakfast Initiative	United Kingdom	Diet	
(Toumbourou, Gregg et al. 2013)	Resilient
Families	Australia	Alcohol	
(Sacchetti, Ceciliani et al. 2013)	Sacchetti	Italy	Physical activity	
(Gomes de Barros, Nahas et al. 2009)	Saude na Boa	Brazil	Physical activity	
(Elinder, Heinemans et al. 2012)	SCIP	Sweden	Physical activity, diet	
(McKay, McBride et al. 2012)	SHAHRP	Northern Ireland	Alcohol	
(Evans, Greenwood et al. 2010)	SMART lunch box	United Kingdom	Diet	
(Sichieri, Paula et al. 2009)	SODA	United Kingdom	Sugar-sweetened beverage consumption	
(Toftager, Christiansen et al. 2014)	SPACE	Denmark	Physical activity	
(Marcus, Nyberg et al. 2009)	STOPP	Sweden	Physical activity, diet	
(Stamm-Balderjahn, Groneberg et al. 2012)	Students in the Hospital	Germany	Smoking	
(Gentile, Welk et al. 2009)	Switch what you Do, View and Chew	United States	Physical activity, diet	
(Rohrbach, Sun et al. 2010)	Towards No Drug abuse (TND)	United States	Smoking, alcohol	
(Yýldýrým, Arundell et al. 2013)	Transform-Us	Melbourne	Physical activity	
(Gabrhelik, Duncan et al. 2012)	Unplugged	Czech Republic	Smoking, alcohol	
(Giles, Kenney et al. 2012)	WATER 	United States	Physical activity, diet	
(Van Lier, Huizink et al. 2009)	Van Lier	Netherlands	Smoking, alcohol	
(Vitoria, Silva et al. 2011)	Vitória	Portugal	Smoking	
(Wen, Fry et al. 2008)	Wen	Australia	Physical activity	
(Balvig et al. 2011)	Ripple effect	Denmark	Tobacco and alcohol	
(Hands et al. 2011)	Hands	Australia	Physical activity	
(Vincus et al. 2010)	Project DARE	US	Tobacco and alcohol	
(West et al. 2008)	Project Northland 	Croatia	Alcohol	
