# Supplementary material for: Socioeconomic gradients in the effects of universal school-based health behaviour interventions: a systematic review of intervention studies
Source: BMC Public Health. 2015 Sep 17;15:907. doi: 10.1186/s12889-015-2244-x (PMC4574356; doi:10.1186/s12889-015-2244-x)
Supplement: Additional file 2: — Search strategy. (DOCX 18 kb) [file 12889_2015_2244_MOESM2_ESM.docx]

**Medline search strategy (subject headings adapted for Embase, Psychinfo)**

1. exp diet/ or hyperphagia/ or dietary fats/

2. ((calori* or fat? or fatty or fizz* or soft* or carbonated* or sweetened or salt* or sugar* or fruit* or veg? or vegetable* or fibre* or fiber* or 5-a-day or five a day or go for 2&5) adj2 (intake or food* or diet* or consum* or meal* or eat* or nutrition or drink* or snack*)).ti,ab.

3. ((poor or over* or unhealthy or health*) adj3 (nutrition or diet* or eat* or meal* or food* or snack* or drink*)).ti,ab.

4. (lunch or breakfast or school meal or vending machine).ti,ab.

5. or/1-4

6. exp exercise/ or physical exertion/ or physical fitness/ or physical endurance/ or fitness/

7. Baseball/ or basketball/ or bicycling/ or cycling/ or boxing/ or dancing/ or football/ or gardening/ or golf/ or gymnastics/ or hockey/ or exp martial arts/ or mountaineering/ or exp racquet sports/ or exp running/ or skating/ or snow sports/ or soccer/ or exp swimming/ or volleyball/ or walking/ or weight lifting/ or wrestling/ or rugby/ or hockey/ or netball/ or basketball/ or physical education/ or sports/

8. (Physical activity or fitness or physical* fit* or physical exert* or exercise or aerobic activit* or sport* or aerobic capacity or active lifestyle* or outdoor activit* or gym* or mvpa).ti,ab.

9. ((fitness or leisure) adj2 (class* or regime* or program* or centre * or center*)).ti,ab.

10. ((fit* or sport* or activ* or exercise or physical exer*) adj3 (lack* or low or no or absen*)).ti,ab.

11. Sedentary lifestyle/ or Video games/

12. gaming.ti,ab.

13. ((view* or watch* or play* or game* or gaming or use* or using or usage) adj2 (television or tv or video* or dvd* or screen or comput* or laptop* or media)).ti,ab.

14. ((screen or sedentary or view*) adj2 (time or hour* or minute*)).ti,ab.

15. ((inactiv* or seden* or indoor*) adj3 (lifestyle* or activit*)).ti,ab.

16. (walk* or cycl* or gym* or danc* or physical educ*).ti,ab.

17. or/6-16

18. Smoking/

19. smoking.ti,ab.

20. ((smok* or tobacco or cigarette* or nicotine) adj3 (addict* or use* or usage or using or intake or consum*)).ti,ab.

21. ((abstain* or abstinence or reduc* or declin* or quit* or stop* or cess* or cease* or cut down or giv* up) adj4 (smok* or tobacco or cigarette* or nicotine)).ti,ab.

22. ((prevent* or uptake or initiation) adj4 (smok* or tobacco or cigarette* or nicotine)).ti,ab.

23. ((smok* or tobacco or cigarette* or nicotine) adj3 prevalence).ti,ab.

24. ((tobacco or cigarette* or nicotine) adj3 (addict* or use* or usage or using or intake or consum*)).ti,ab.

25. or/18-24

26. exp Drinking Behavior/

27. exp Alcohol-Related Disorders/

28. ((alcohol* or ethanol or beer or cider or wine or spirit* or alcopop *) adj3 (use* or usage* or using or intake or consum* or drink* or misus* or abus*)).ti,ab.

29. ((alcohol* or drink* or ethanol) adj3 (excess* or binge* or binging or intoxicat* or poison* or risk* or depend*)).ti,ab.

30. (units adj3 (week or alcohol)).ti,ab.

31. or/26-30

32. Obesity/ or Weight Gain/ or Weight Loss/

33. (obes* or weight gain or weight loss).ti,ab.

| 34. (overweight or over weight or overeat$ or over eat$).ti,ab.  35. ((weight or bmi or body mass index) adj2 (gain or loss or change)).ti,ab |  |
| --- | --- |
| 36. or/32-35 |  |

37. (randomized controlled trial or controlled clinical trial).pt.

38. (randomi#ed or placebo or randomly).ab.

39. trial.ti.

40. quasi experiment*.ti,ab.

41. clinical trials as topic.sh.

42. or/37-41

43. exp Schools/

44. (school* or student* or pupil*).tw.

45. ((young* or adolesc* or teen* or primary or infant or minor* or boy* or girl* or youth* or secondary) adj3 (educat* or school* or highschool* or high?school*)).mp.

46. or/43-45

47. or/5,17,25,31,36

48. and/42,46-47

49. limit 48 to human

50. limit 49 to english

51. limit 50 to yr=2008-2014

**ProQuest search strategy (Sociological abstracts, ASSIA, ERIC, BEI)**

((su,ti,ab(child*4 OR adolesc*4 AND school*9)) AND (su(randomized controlled trial) OR ti,ab(randomly OR placebo OR randomi?ed OR quasi?experiment*2 OR trial))) AND (((su,ti,ab (exercise OR physical fitness OR fitness OR physical activity OR sport*4 OR sedentary lifestyle OR video games)) OR ab((view*3 OR watch*3 OR play*3 OR gam*3 OR use OR using OR usage OR time) NEAR/2 (television OR tv OR video*5 OR dvd*2 OR screen OR compute*2 OR laptop*1 OR media)) OR ab(walk*3 OR cycl*3 OR gym*7 OR danc*3 OR physical educ*5 OR inactiv*3 OR seden*6 OR leisure OR activ*3)) OR ((su(diet) OR su(hyperphagia) OR su(dietary fats)) OR ab(nutrition OR diet*3 OR eat*3 OR meal*1 OR food*1 OR snack*3 OR drink*3 OR calori* OR fat? OR fatty OR fizz*1 OR soft* OR carbonated OR sweetened OR salt*2 OR sugar* OR fruit*1 OR veg* OR fibre* OR fiber* OR lunch OR breakfast OR school meal OR vending machine)) OR (su(drinking behavio?r) OR su(alcohol-related disorders) OR ab((alcohol*3 OR ethanol OR drink*3 OR beer*1 OR cider OR wine OR spirit*1))) OR ((su (smoking)) OR ab(smoking) OR ab((smok*3 OR tobacco OR cigarette*1 OR nicotine))))
